# MGA-UNet: A Frequency-Aware Multi-Scale Mamba U-Net for Medical Image Segmentation

**DOI:** 10.3390/s26175416

**Published:** 2026-08-27

**Authors:** Shuaikang Qiu, Xuan Wang, Kaile Su, Yongchao Song, Qiang Zheng, Zhenbo Cao

**Affiliations:** School of Computer and Control Engineering, Yantai University, Yantai 264005, China; qiushuaikang@s.ytu.edu.cn (S.Q.); ycsong@ytu.edu.cn (Y.S.);

**Keywords:** medical image segmentation, wavelet transform, sparse attention, Mamba

## Abstract

Medical image segmentation is a critical task in computer-assisted diagnosis, but accurate delineation remains challenging in sensor-captured dermoscopic and endoscopic images because of low contrast, blurred boundaries, acquisition artifacts, and large appearance variations. Although CNN-based methods are effective in capturing local texture and boundary cues, they often struggle to explicitly model long-range dependencies and global structural relationships. Transformer-based architectures can capture global context, but their self-attention mechanism may become computationally costly when processing high-resolution feature maps. To address these challenges, we propose MGA-UNet, a frequency-aware multi-scale encoder–decoder segmentation framework that integrates wavelet-based frequency decomposition with Mamba-based long-range dependency modelling. Specifically, the Wavelet-Mamba feature extraction backbone (WMB) decomposes features into low- and high-frequency components to enhance boundary-aware representation, the Gated Multi-scale Aggregation Module (GMAM) aggregates parallel multi-scale encoder features and applies a content-dependent gate to the fused response, and the Adaptive Sparse Attention Module (ASAM) refines bottleneck representations with sparse attention for global semantic modelling. Across three independent runs with random seeds 42, 123, and 2026, MGA-UNet achieves mean Dice Similarity Coefficients of 88.92±0.04%, 88.01±0.07%, and 85.91±0.04% on ISIC2018, ISIC2017, and Kvasir-SEG, respectively. These results demonstrate competitive segmentation performance among the compared representative CNN-based, Transformer-based, and Mamba-based methods, including the recent H-VMUNet baseline. These results indicate that frequency-domain decomposition and state-space modelling can complement each other for accurate medical image segmentation, particularly in images with ambiguous boundaries and complex background interference.

## 1. Introduction

Medical image segmentation has gained substantial attention in recent years. Its objective is to accurately localize and delineate anatomical structures or pathological regions of interest from images acquired by clinical sensing systems. Unlike natural images captured under relatively consistent lighting, dermoscopic and endoscopic images are generated through modality-specific acquisition pipelines that may introduce illumination variation, heterogeneous noise, specular reflection, and other sensor- or modality-dependent artifacts. For instance, in the polyp segmentation dataset Kvasir-SEG, lesion regions frequently exhibit color and texture similarities to surrounding normal tissues, resulting in indistinct boundaries. In the skin lesion segmentation datasets ISIC2017 and ISIC2018, severe hair occlusion may obscure lesion margins, making lesion regions increasingly ambiguous and difficult to delineate accurately. These characteristics markedly increase the difficulty of accurate segmentation [1,2].

Convolutional neural networks have driven significant progress in this field, while U-Net [3] established the classic encoder–decoder architectural paradigm. Numerous subsequent variants have continued to improve performance by enhancing the backbone network, the bottleneck structure, and skip connections. At the heart of this series of successes lies U-Net’s ability to efficiently fuse semantic information with spatial details. Building on this foundation, many variants have been proposed, such as U-Net++ [4], RA-UNet [5], CPFNet [6], and ResUNet [7], which introduce architectural refinements to enhance feature representation. Despite these improvements, the intrinsic locality and gradually expanding receptive fields of CNNs limit their ability to capture long-range contextual dependencies in complex medical images [8]. Transformer-based methods, introduced from NLP, alleviate this limitation through global self-attention but suffer from quadratic complexity, making them difficult to apply to high-resolution medical images [9]. The recently proposed Mamba [10] model provides an appealing alternative, offering linear-time sequence modeling while retaining strong long-range representation capability. However, when applied to medical imaging, Mamba may still inadequately preserve high-frequency structural details and adapt to low-contrast or texture-intensive scenarios [11,12]. Moreover, traditional encoder–decoder frameworks often exhibit semantic inconsistency in skip connections. Directly merging shallow high-frequency features with deep semantic representations may introduce noise and cause misidentification of small lesions, while single-scale skip connections limit the ability to handle diverse anatomical sizes and complex appearance variations.

**Figure 1:**
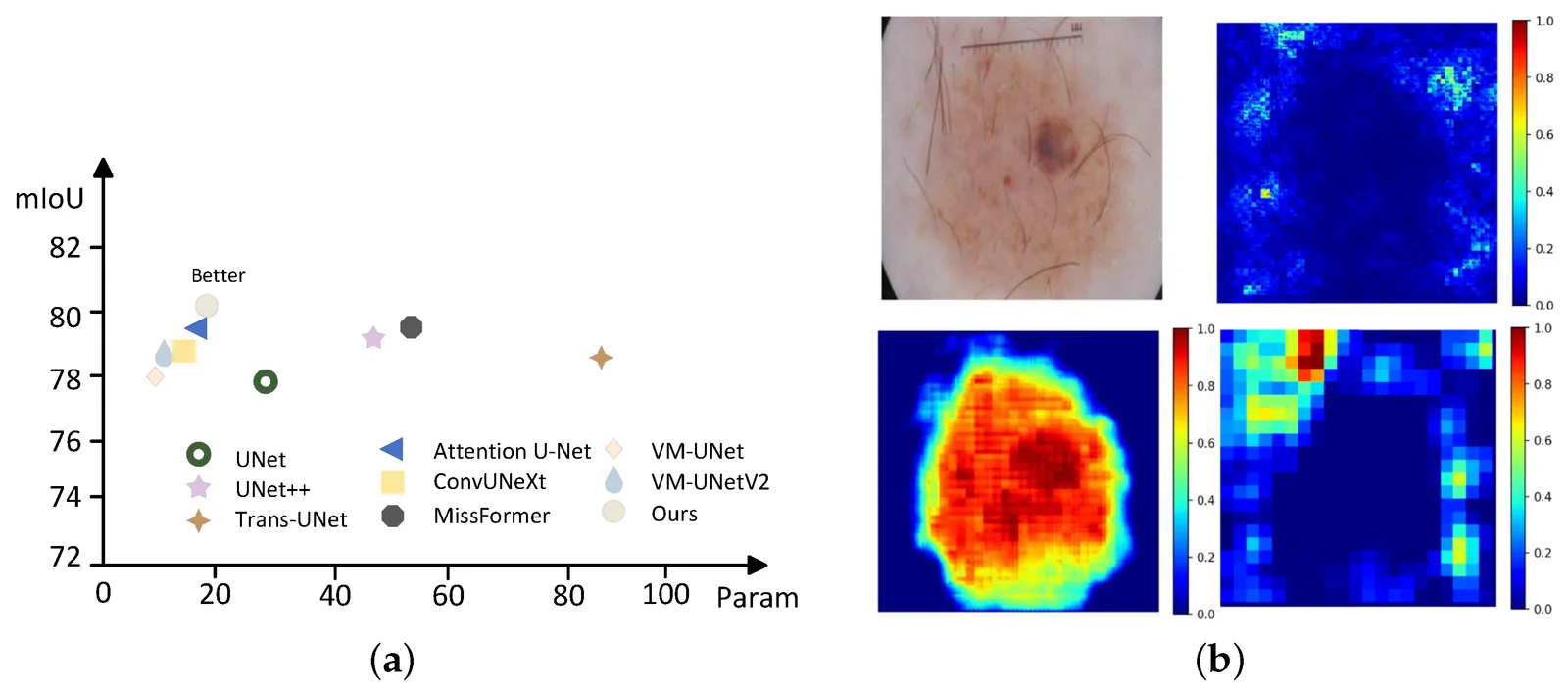
(**a**) Comparison of parameter efficiency and mIoU among different models. (**b**) Qualitative visualization of model interpretability, showing the input image, input gradient, Grad-CAM, and occlusion-based importance map.

In U-shaped architectures, the bottleneck region serves as the central hub for information exchange between the encoder and decoder [13]. The encoder progressively compresses spatial details into a high-dimensional yet low-resolution semantic representation, while the decoder must gradually restore fine-grained spatial information during upsampling. Consequently, the bottleneck must strike an effective balance between deep semantic abstraction and preservation of intricate local structures—its modeling capacity directly governs boundary delineation accuracy and the overall fidelity of structural reconstruction [14]. As illustrated in Figure 1b, the input-gradient, Grad-CAM, and occlusion-based importance maps provide complementary views of the regions contributing to the prediction. The activation responses are primarily concentrated on the target region and its surrounding boundaries, indicating that MGA-UNet relies on both lesion-level semantic information and boundary-sensitive features. These qualitative observations are consistent with the intended global-local representation design of the proposed framework. To address the aforementioned challenges, this paper proposes MGA-UNet, a frequency-aware and adaptive global-local representation framework for medical image segmentation. Unlike existing approaches that directly combine individual architectural components, MGA-UNet is designed according to the heterogeneous characteristics of medical image features, including global anatomical structures, local boundary details, and multi-scale semantic representations. Specifically, the Wavelet-Mamba feature extraction backbone (WMB) introduces a frequency-specific heterogeneous modeling strategy, where low-frequency components containing global structural information are processed by Mamba-based SS2D for long-range dependency modeling, while high-frequency components containing boundary and texture information are enhanced by depthwise convolution to preserve local details. The gated multi-scale aggregation module (GMAM) differs from conventional multi-scale fusion strategies by introducing a learnable content-dependent gate, which adaptively regulates cross-scale feature interactions and reduces semantic discrepancies between encoder and decoder representations. Furthermore, the adaptive sparse attention module (ASAM) performs adaptive sparse semantic refinement at the bottleneck stage, dynamically selecting informative features instead of relying on fixed sparse patterns. Through these complementary designs, MGA-UNet achieves an effective balance between global dependency modeling, local detail preservation, and computational efficiency. Extensive experimental results demonstrate that MGA-UNet provides competitive segmentation performance on the three challenging datasets: ISIC2017, ISIC2018, and Kvasir-SEG. The main contributions of this work are summarized as follows:

- We propose MGA-UNet, a frequency-aware global–local representation framework for medical image segmentation. The framework integrates frequency-specific feature extraction, hierarchical multi-scale aggregation, and bottleneck semantic refinement within a unified encoder–decoder architecture.
- We design the Wavelet-Mamba Backbone, which employs SS2D to model long-range dependencies in low-frequency structural features and depthwise convolution to preserve localized boundary and texture information in high-frequency features. This heterogeneous processing strategy enables complementary global structure modeling and local detail preservation.
- We introduce the Gated Multi-scale Aggregation Module to align and aggregate hierarchical encoder features. By applying content-dependent gating together with spatial and channel refinement, GMAM reduces semantic discrepancies among multi-resolution features during encoder–decoder feature interaction.
- We develop the Adaptive Sparse Attention Module for bottleneck feature refinement. ASAM combines adaptive sparse self-attention with an adaptive gated feed-forward network to emphasize informative semantic interactions and enhance global representation. Experiments on ISIC2017, ISIC2018, and Kvasir-SEG demonstrate the effectiveness and competitive performance of the proposed framework.

## 2. Related Work

### 2.1. Medical Image Segmentation

Medical image segmentation is a key component in disease diagnosis and treatment planning. It aims to accurately delineate organs or lesions from medical images such as CT and MRI. Deep learning has significantly advanced this field. In particular, FCNs [15] laid an important foundation for medical image segmentation. U-Net further improved performance with its symmetric encoder–decoder structure and skip connections. It has remained a dominant method for a long time. Inspired by U-Net, many variants have been proposed to address specific challenges. U-Net++ [4] introduces dense skip connections between the encoder and decoder. It performs progressive feature fusion across different resolutions. This design reduces feature redundancy and improves multi-scale representation. Attention U-Net [16] incorporates attention mechanisms into skip connections. It enables the network to focus on target regions while suppressing irrelevant responses. In recent years, Transformer-based methods have shown strong potential due to their global modeling capability. MISSFormer [17] introduces a ReMixFFN module to integrate local and global information. This improves multi-scale feature representation. RTFormer [18] adopts a dual-resolution architecture. A high-resolution branch guides a low-resolution branch to learn global context. TransFuse [19] combines Transformers and convolutional neural networks in a parallel manner. It captures both global dependencies and fine-grained spatial details. Multimodal information fusion has also received increasing attention in clinically oriented segmentation. Li et al. [20] proposed SymUnet-DynCFC to integrate complementary MRI information for robust cartilage segmentation and clinically relevant knee osteoarthritis assessment, highlighting the value of multimodal feature integration in medical image analysis. In addition, SAT-Net [21] proposes a structure-aware Transformer attention fusion network for enhancing low-quality retinal fundus images. The method captures global spatial dependencies by combining window-wise and channel-wise self-attention, uses cross-quality knowledge distillation to preserve fine-grained topological structures in a lightweight network, and introduces a structure-aware multi-scale loss to retain key vascular details. More recently, Li et al. [22] proposed SwMrNet for robust multi-target tissue segmentation in knee MRI. By combining global contextual modeling with multi-scale local feature refinement, the method improves the representation of complex anatomical structures and further demonstrates the importance of robust global-local feature modeling in clinically oriented medical image segmentation.

### 2.2. Selective State Space Models

State space models (SSMs) were originally designed for efficient sequence modelling in temporal and language tasks. In recent years, they have gained prominence in the field of computer vision due to their linear computational complexity and powerful ability to capture long-range dependencies. Mamba leverages selective state space modeling to enable linear-time sequence processing, providing an efficient alternative to attention-based architectures for long-range dependency modeling in vision tasks. Compared with Transformers that rely on global self-attention, Mamba provides more efficient and stable spatiotemporal modeling, alleviating the computational bottleneck present in high-resolution vision tasks. As a result, Mamba has been rapidly adopted in various computer vision domains. Gu et al. [10] demonstrated its strong long-range dependency modeling capability in sequential modeling tasks. Liu et al. [23] proposed VMamba, which extends selective state-space modeling to two-dimensional visual data through a 2D selective scanning mechanism and achieves strong performance in semantic segmentation and object detection. To further enhance segmentation performance, Wu et al. [24] proposed H-VMUNet, a high-order vision Mamba U-Net for medical image segmentation. H-VMUNet employs high-order 2D selective scanning (H-SS2D) instead of the original SS2D, reducing redundant information via high-order interactions. Swin-UMamba [25] combines the sequence modelling strengths of Mamba with hierarchical feature extraction by incorporating the VSS module, thereby effectively enhancing the efficiency of semantic representation. VM-UNet [26] embeds Mamba blocks in both the encoder and decoder, thereby enabling efficient modelling of long-range dependencies within a U-shaped architecture and achieving competitive performance. U-Mamba [27] further enhances the stability and convergence of high-resolution medical images by introducing a hierarchical SSM design and hybrid residual connections. Yang et al. [28] extend Mamba to multimodal reference image segmentation, proposing ReMamber and its Mamba Twister block, which achieve efficient vision-language feature fusion through channel and spatial distortion mechanisms. In addition, CaVMamba [29] combines the powerful local feature extraction capability of convolutions with the global modeling advantages of VMamba, employing a unique sandwich structure to achieve deep fusion of the two features, and introduces a dynamic feature fusion module to selectively enhance multi-scale features.

### 2.3. Wavelet Transform

In medical image processing, spatial-domain analysis, while intuitive, is inherently limited. Critical visual cues such as texture, edges, noise characteristics, and structural artifacts are often inextricably intertwined within the spatial representation. In contrast, frequency-domain transformations offer complementary and insightful perspectives on the underlying signal. Xu et al. [30] introduced the Fourier Analysis Network (FAN) as a visual backbone, which explicitly encodes periodic patterns in neural networks through Fourier series. This approach provides a principled mechanism for directly modeling periodicity from data. However, FAN primarily emphasizes global frequency representations and lacks fine-grained modeling of local spatial details and multi-scale structures. Consequently, its effectiveness is limited in medical image segmentation tasks that involve blurred boundaries and substantial scale variations. Although the Fourier Transform decomposes an image into sinusoidal components and effectively reveals its global frequency characteristics, its inability to provide spatial localization significantly restricts its applicability to complex, spatially varying medical images. The Wavelet Transform addresses this limitation by employing scalable wavelet bases with adaptive resolution, enabling the simultaneous extraction of coarse structural information and fine-grained texture details [31,32]. This multi-resolution property makes wavelet-based representations particularly well-suited for medical images, where structures of interest often exhibit strong scale variability and localized texture patterns.

### 2.4. Self-Attention-Based Models

Self-attention mechanisms are increasingly used in medical image segmentation because they can capture long-range dependencies beyond the fixed receptive fields of convolutional networks [33]. However, their quadratic computational and memory cost makes them difficult to apply to high-resolution dense prediction tasks. To reduce this burden, hybrid models such as TransUNet [34] apply self-attention only to low-resolution encoder features. This design preserves global context while lowering computational cost. Window-based methods further improve efficiency. For example, the Swin Transformer [35] uses shifted windows to approximate global interactions with linear complexity. Based on this idea, Swin-UNet [36] builds a U-shaped architecture entirely on self-attention and achieves strong performance. Wen et al. [37] proposed RetiNeXt, a lightweight network for retinal vessel segmentation. It extracts global topological features and enhances fine vessel details through a frequency-domain module. It also maintains structural continuity using global feature extraction blocks and improves low-contrast vessel segmentation with the SimAM attention mechanism. Despite these advances, self-attention methods still have limitations in clinical scenarios. They require high memory and often rely on local windows, which can weaken the modelling of fine boundaries [17]. Recent work on sparse and linear attention improves scalability. However, balancing global context modelling with fine-grained representation of small or low-contrast structures remains an open problem.

By seamlessly integrating the wavelet transform with Mamba, we propose a novel encoder–decoder segmentation framework, termed MGA-UNet. This architecture is specifically designed to address key challenges in medical images, including appearance variations, inter-individual differences, and blurred boundaries. Specifically, we introduce a gated multi-scale fusion mechanism to enable efficient multi-scale feature interaction, and integrate an ASAM module at the bottleneck stage to enhance the model’s ability to accurately focus on critical features in low-contrast regions.

## 3. Method

### 3.1. Preliminaries

**Figure 2:**
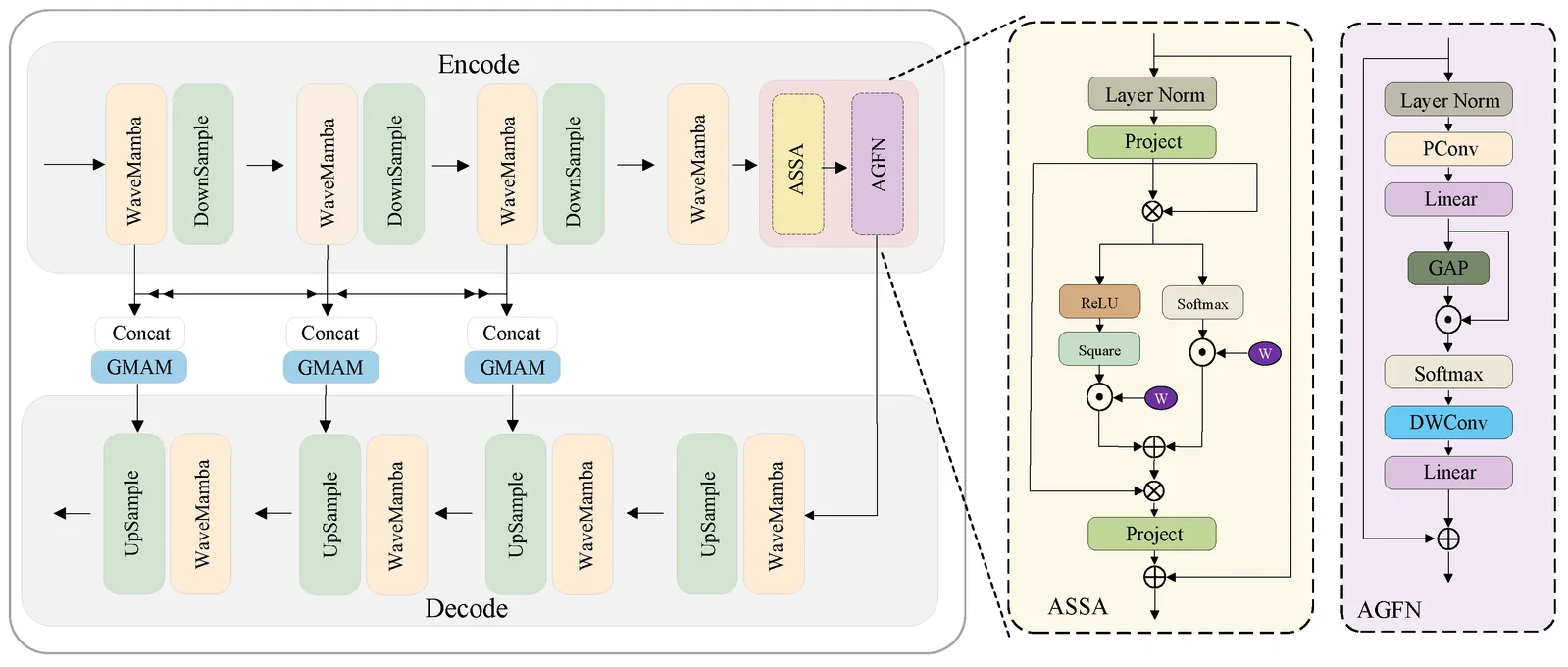
Overall architecture of the proposed MGA-UNet. The proposed MGA-UNet adopts an encoder–decoder architecture. In the encoder, wavelet transform and Mamba are integrated to extract complementary local and global features. These representations are then progressively upsampled to the original image resolution to produce the final segmentation mask.

As shown in Figure 2, MGA-UNet adopts a symmetric encoder–decoder architecture specifically designed for medical image segmentation. The encoder extracts multi-resolution hierarchical features in a step-by-step manner, while the decoder progressively restores fine spatial details through upsampling; both the encoder and decoder consist of four stages, achieving a good balance between expressive power and computational efficiency. In the encoding stage, the input is first processed by the Wavelet-Mamba feature extraction backbone, which performs frequency decomposition and feature modeling at multiple hierarchical levels. The resulting multi-level features are subsequently aligned and adaptively fused by the Gated Multi-scale Aggregation Module. GMAM consists of a gated multi-scale fusion submodule, a Spatial Attention Module (SAM), and a Channel Attention Module (CAM), which jointly integrate spatial details and semantic information from different resolutions. At the bottleneck, the ASAM is introduced to further refine the low-resolution deep features and enhance global semantic modeling. ASAM comprises an ASSA unit and an Adaptive Gated Feed-forward Network . Finally, the decoder progressively upsamples the refined features, restores the spatial resolution, and produces the final segmentation result.

### 3.2. WMB Backbone

**Figure 3:**
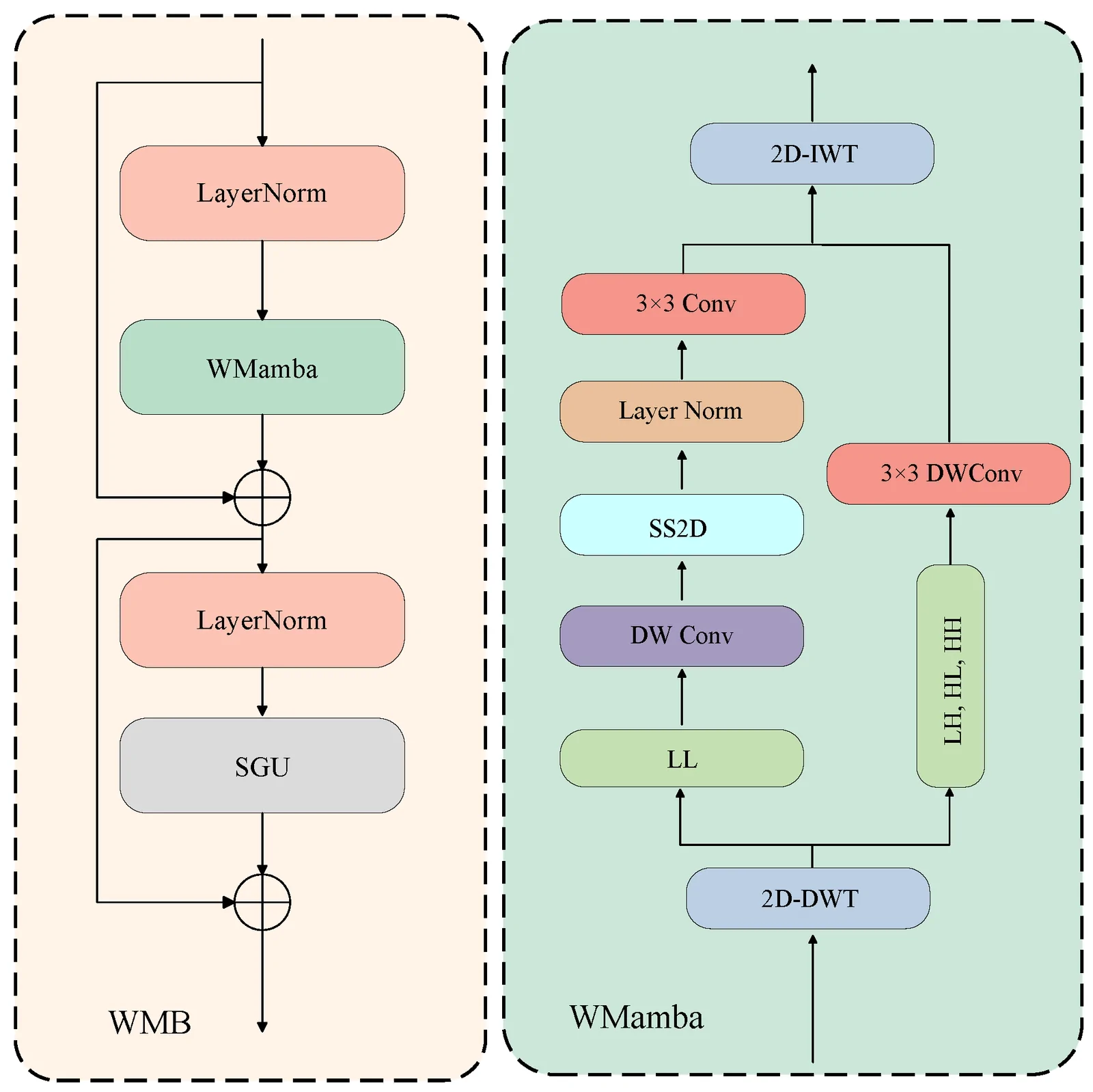
Detailed architecture of the Wavelet-Mamba Block (WMB). The input feature is decomposed into low- and high-frequency sub-bands by DWT. The low-frequency component is processed by SS2D for long-range contextual modeling, while the high-frequency components are enhanced using depthwise convolution. The processed sub-bands are reconstructed by IWT, followed by an SGU for spatial feature refinement.

Let $X_{l}\in R^{C_{l}\times H_{l}\times W_{l}}$ denote the input feature of the *l*-th encoder stage. The Wavelet-Mamba feature extraction backbone (WMB) decomposes $X_{l}$ into one low-frequency sub-band and three high-frequency sub-bands, models them with branch-specific operators, and reconstructs the spatial feature by inverse wavelet transform. As shown in Figure 3, WMB contains four sequential parts: DWT-based frequency decomposition, low-frequency Mamba modeling, high-frequency depthwise convolution, and IWT-based feature reconstruction followed by spatial gating.

The assignment of different operators to the low- and high-frequency branches is motivated by the distinct spatial characteristics of the wavelet sub-bands. The low-frequency LL component retains coarse-scale anatomical structures, slowly varying intensity patterns, and broad spatial relationships. Preserving the consistency of these structures requires information exchange beyond a limited local neighborhood; therefore, SS2D is employed to propagate contextual information along two-dimensional scanning paths and model long-range spatial dependencies with linear complexity. In contrast, the LH, HL, and HH components primarily represent localized intensity transitions, directional edges, and fine texture variations. These high-frequency responses depend mainly on neighboring pixels and require accurate spatial localization rather than extensive global interaction. Depthwise convolution is therefore used to exploit its local inductive bias and enhance each channel efficiently while avoiding the additional computational cost that would result from applying SS2D to all three high-frequency branches. Consequently, this heterogeneous assignment allows WMB to model global structural context in the low-frequency branch while preserving fine boundary details in the high-frequency branches.

Formally, the 2D DWT is applied independently to each channel along the height and width dimensions. Unless otherwise stated, all reported experiments use the Haar wavelet, and the sub-band order is fixed as (LL,LH,HL,HH). Because the input images are resized to 256×256 and the encoder maintains even spatial dimensions at all four stages, no odd-size padding is required in the reported experiments:

(1) $$\left(X_{l}^{LL},X_{l}^{LH},X_{l}^{HL},X_{l}^{HH}\right)=\mathrm{DWT}\left(X_{l}\right),$$

where

(2) $$X_{l}^{LL},X_{l}^{LH},X_{l}^{HL},X_{l}^{HH}\in R^{C_{l}\times \frac{H_{l}}{2}\times \frac{W_{l}}{2}}.$$

Here, $X_{l}^{LL}$ preserves low-frequency structural and contour information, whereas $X_{l}^{LH}$, $X_{l}^{HL}$, and $X_{l}^{HH}$ encode high-frequency details along different directional responses. The high-frequency sub-bands are channel-wise concatenated as

(3) $$X_{l}^{H}=\mathrm{Concat}\left(X_{l}^{LH},X_{l}^{HL},X_{l}^{HH}\right)\in R^{3C_{l}\times \frac{H_{l}}{2}\times \frac{W_{l}}{2}}.$$

The low- and high-frequency branches are then processed separately:

(4) $${\tilde{X}}_{l}^{LL}=\mathrm{SS}2D\left(X_{l}^{LL}\right),\;{\tilde{X}}_{l}^{H}=\mathrm{DWConv}\left(X_{l}^{H}\right),$$

where ${\tilde{X}}_{l}^{LL}\in R^{C_{l}\times \frac{H_{l}}{2}\times \frac{W_{l}}{2}}$ and ${\tilde{X}}_{l}^{H}\in R^{3C_{l}\times \frac{H_{l}}{2}\times \frac{W_{l}}{2}}$. Before reconstruction, the processed high-frequency tensor is split channel-wise into three directional sub-bands:

(5) $$\left({\tilde{X}}_{l}^{LH},{\tilde{X}}_{l}^{HL},{\tilde{X}}_{l}^{HH}\right)={\mathrm{Split}}_{C}\left({\tilde{X}}_{l}^{H}\right),$$

where each sub-band belongs to $R^{C_{l}\times \frac{H_{l}}{2}\times \frac{W_{l}}{2}}$. The four processed sub-bands are then provided explicitly to IWT:

(6) $${\hat{X}}_{l}=\mathrm{IWT}\left({\tilde{X}}_{l}^{LL},{\tilde{X}}_{l}^{LH},{\tilde{X}}_{l}^{HL},{\tilde{X}}_{l}^{HH}\right)\in R^{C_{l}\times H_{l}\times W_{l}}.$$

Finally, LayerNorm and the Spatial Gating Unit are used to obtain the WMB output:

(7) $$F_{l}=\mathrm{SGU}\left(\mathrm{LN}({\hat{X}}_{l})\right)\in R^{C_{l}\times H_{l}\times W_{l}}.$$

In this way, WMB preserves the dimensional consistency required by the encoder while explicitly separating structural low-frequency information from boundary-sensitive high-frequency details.

### 3.3. GMAM Module

**Figure 4:**
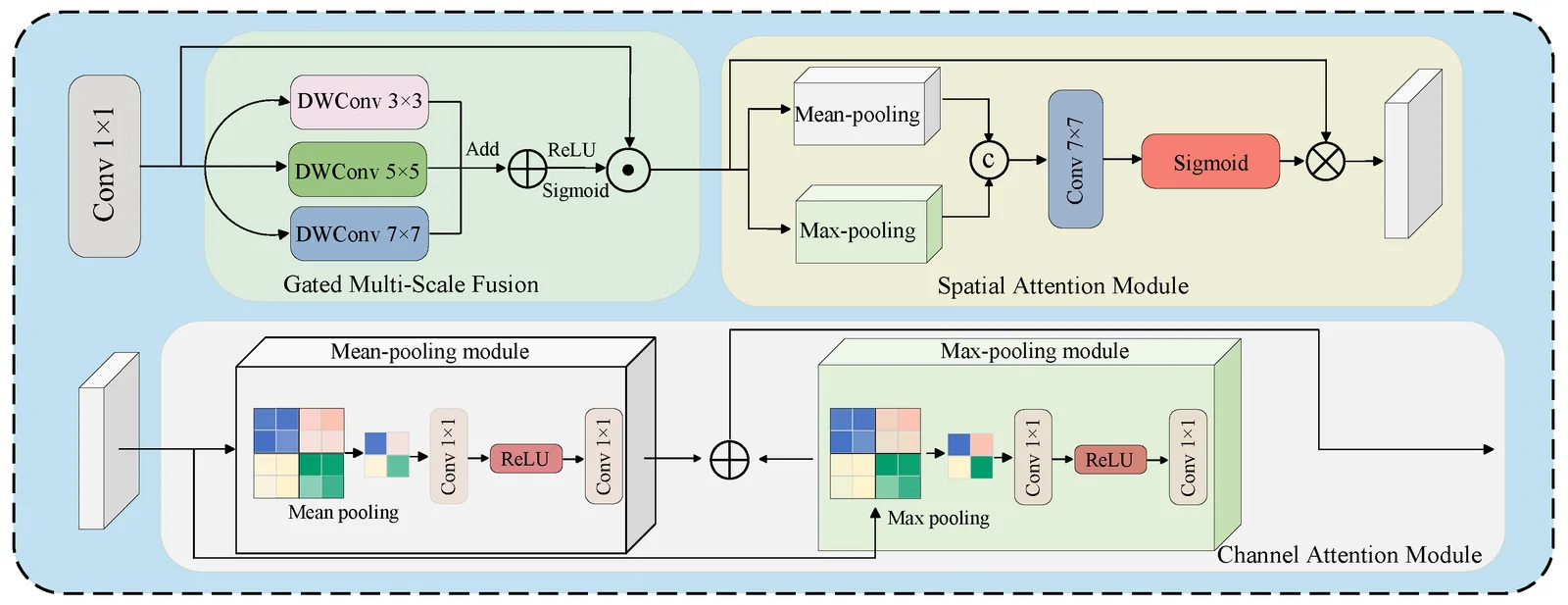
Detailed architecture of the GMAM module: an end-to-end information flow comprising a gated multi-scale fusion submodule, a spatial attention submodule, and a channel attention submodule.

The Gated Multi-scale Aggregation Module (GMAM) is designed to fuse hierarchical encoder features and refine the fused representation from both spatial and channel dimensions. GMAM is composed of three parts (Figure 4): multi-scale feature alignment, parallel gated multi-scale fusion (GMSF), and sequential SAM–CAM refinement.

Let ${\left\{F_{i}\right\}}_{i=1}^{L}$ denote the set of encoder features to be fused, where $F_{i}\in R^{C_{i}\times H_{i}\times W_{i}}$. Before concatenation, each feature is first resized to the target spatial resolution $\left(H_{t},W_{t}\right)$ and projected to the same channel dimension $C_{t}$:

(8) $${\bar{F}}_{i}=\phi_{i}\left(\mathrm{Resize}(F_{i};H_{t},W_{t})\right)\in R^{C_{t}\times H_{t}\times W_{t}},\;i=1,2,\ldots ,L,$$

where Resize(·) denotes bilinear interpolation for upsampling or pooling/strided interpolation for downsampling, and $\phi_{i}$ is a 1×1 convolution used for channel alignment. The aligned features are concatenated and compressed by a 1×1 convolution to obtain the input feature of GMSF:

(9) $$F_{\mathrm{concat}}={\mathrm{Conv}}_{1\times 1}\left(\mathrm{Concat}({\bar{F}}_{1},{\bar{F}}_{2},\ldots ,{\bar{F}}_{L})\right)\in R^{C_{t}\times H_{t}\times W_{t}}.$$

In this way, the spatial sizes of all multi-level features are aligned before concatenation, and the channel dimension of $F_{\mathrm{concat}}$ is unified to $C_{t}$.

The GMSF process is defined as follows:

(10) $$Y_{1i}={\mathrm{DWConv}}_{k_{i}\times k_{i}}\left(F_{\mathrm{concat}}\right),\;k_{i}\in \left\{3,5,7\right\},\;i=1,2,3,$$

(11) $$Y_{\Sigma }=\sum_{i=1}^{3}Y_{1i},\;G_{\mathrm{agg}}=\sigma \left({\mathrm{Conv}}_{1\times 1}\left(Y_{\Sigma }\right)\right),$$

(12) $$Y_{\mathrm{GMSF}}=G_{\mathrm{agg}}⊙Y_{\Sigma },$$

(13) $$Y_{l}=\mathrm{CAM}\left(\mathrm{SAM}\left(Y_{\mathrm{GMSF}}\right)\right).$$

Here, $Y_{1i}\in R^{C_{t}\times H_{t}\times W_{t}}$ denotes the response of the *i*-th parallel depthwise convolution branch, $Y_{\Sigma }$ is their aggregated multi-scale response, and $G_{\mathrm{agg}}\in R^{C_{t}\times H_{t}\times W_{t}}$ is a content-dependent gate generated from the aggregated feature. The gate is generated from the aggregated representation rather than assigned separately to each branch, and it reweights the fused response element-wise. $Y_{\mathrm{GMSF}}\in R^{C_{t}\times H_{t}\times W_{t}}$ is the gated fusion output, $Y_{l}\in R^{C_{t}\times H_{t}\times W_{t}}$ is the final output of GMAM, ⊙ denotes element-wise multiplication, and σ is the sigmoid activation.

For completeness, the SAM and CAM operations are explicitly defined as feature reweighting operations rather than standalone attention masks. Given an input feature $X\in R^{C\times H\times W}$, SAM is computed as

(14) $$M_{\mathrm{SAM}}\left(X\right)=\sigma \left({\mathrm{Conv}}_{7\times 7}\left(\mathrm{Concat}\left({\mathrm{Avg}}_{c}\left(X\right),{\mathrm{Max}}_{c}\left(X\right)\right)\right)\right)\in R^{1\times H\times W},$$

(15) $$\mathrm{SAM}\left(X\right)=X⊙M_{\mathrm{SAM}}\left(X\right).$$

Similarly, CAM is computed as

(16) $$M_{\mathrm{CAM}}\left(X\right)=\sigma \left(\mathrm{MLP}\left(\mathrm{GAP}(X)\right)+\mathrm{MLP}\left(\mathrm{GMP}(X)\right)\right)\in R^{C\times 1\times 1},$$

(17) $$\mathrm{CAM}\left(X\right)=X⊙M_{\mathrm{CAM}}\left(X\right).$$

In the above formulas, ${\mathrm{Avg}}_{c}\left(\cdot \right)$ and ${\mathrm{Max}}_{c}\left(\cdot \right)$ denote channel-wise average and maximum operations, while GAP(·) and GMP(·) denote global average and max pooling over the spatial dimensions.

### 3.4. ASAM Module

At the bottleneck stage of the U-shaped architecture, feature maps undergo multiple aggressive downsampling operations. This reduces spatial resolution and may weaken fine-grained structural cues. To enhance global semantic modeling under this low-resolution setting, we propose the Adaptive Sparse Attention Module (ASAM). ASAM consists of two sequential components: a top-*k* Adaptive Sparse Self-Attention (ASSA) unit for long-range dependency modeling and an Adaptive Gated Feed-forward Network (AGFN) for local channel refinement. The overall data flow follows a pre-normalization residual design. The attention residual is first computed as

(18) Z=X+ASSA(LN(X)),

after which AGFN produces the final ASAM output, as defined below.

Given the normalized bottleneck feature $X_{n}=\mathrm{LN}\left(X\right)\in R^{C\times H\times W}$, we first flatten its spatial dimensions to obtain ${\bar{X}}_{n}\in R^{N\times C}$, where N=HW. The query, key, and value matrices are computed as:

(19) $$Q={\bar{X}}_{n}W_{q},\;K={\bar{X}}_{n}W_{k},\;V={\bar{X}}_{n}W_{v},$$

where $W_{q},W_{k},W_{v}\in R^{C\times d}$ and $Q,K,V\in R^{N\times d}$. For notational simplicity, the head index is omitted; in the multi-head implementation, the following operations are performed independently within each head. The attention logits are defined as

(20) $$S=\frac{QK^{T}}{\sqrt{d}}+B,$$

where *B* is the relative positional bias. To explicitly impose sparse attention interactions, ASSA keeps only the top-*k* keys with the largest affinity scores for each query token:

(21) $$\Omega_{i}=\mathrm{TopK}\left(S_{i,:},k\right),\;k=\max\left(k_{\min},\left\lceil \rho N\right\rceil \right),$$

where ρ∈(0,1] denotes a fixed sparse ratio and $k_{\min}$ is used to avoid over-sparsification when *N* is small. In this work, “adaptive” refers to content-dependent index selection: the retained set $\Omega_{i}$ is recomputed from the current affinity scores for every query token and independently within each attention head. Thus, the selected spatial positions vary with the input features, whereas ρ and $k_{\min}$ remain fixed hyperparameters and the sparsity ratio itself is not learned. The top-*k* sparse mask is defined as

(22) $$M_{ij}=\left\{\begin{array}{cc} 0, & j\in \Omega_{i},\\ -\infty , & j\notin \Omega_{i}, \end{array}\right.$$

and the sparse attention map is computed by applying Softmax only over the retained key positions:

(23) $$A_{\mathrm{top}k,ij}=\frac{\exp(S_{ij}+M_{ij})}{\sum_{t=1}^{N}\exp\left(S_{it}+M_{it}\right)}.$$

The ASSA output is then obtained as

(24) $$F_{\mathrm{ASSA}}=\mathrm{Proj}\left(A_{\mathrm{top}k}V\right)\in R^{N\times C},$$

which is reshaped back to $R^{C\times H\times W}$ and added to the input through a residual connection as defined above. In this formulation, only the *k* most relevant key positions contribute to each query output, thereby suppressing non-salient interactions in the bottleneck representation.

To complement sparse self-attention with local channel-wise refinement, AGFN is applied to the normalized residual feature LN(Z). It is defined as

(25) $$U=\mathrm{DWConv}\left({\mathrm{PWConv}}_{1}\left(\mathrm{LN}\left(Z\right)\right)\right),$$

(26) $$G=\sigma \left({\mathrm{PWConv}}_{g}\left(\mathrm{GAP}(U)\right)\right),$$

(27) $$F_{\mathrm{AGFN}}={\mathrm{PWConv}}_{2}\left(U⊙G\right),$$

(28) $$Y_{\mathrm{ASAM}}=Z+F_{\mathrm{AGFN}}.$$

Here $U\in R^{C\times H\times W}$ denotes the locally enhanced feature, $G\in R^{C\times 1\times 1}$ denotes the adaptive channel gate, ⊙ denotes element-wise multiplication, PWConv denotes point-wise convolution, DWConv denotes depthwise convolution, and GAP denotes global average pooling. This formulation explicitly defines ASAM as top-*k* ASSA followed by AGFN, clarifies the pre-normalization residual data flow, and aligns the residual connections and normalization operations with the module design.

The purpose of the top-*k* selection is to suppress non-salient token interactions and concentrate the bottleneck representation on the most relevant content-dependent dependencies. ASAM is applied only at the low-resolution bottleneck stage; accordingly, we describe it as a representation-level sparsification mechanism rather than claiming asymptotically linear attention complexity.

### 3.5. Loss Function

To alleviate the severe class imbalance problem in medical image segmentation, in which foreground structures such as lesions or polyps account for only a small fraction of the image, we adopt a hybrid loss function that integrates Binary Cross-Entropy loss with Dice loss. Both terms are assigned equal weights to construct the BCE–Dice loss, enabling the model to simultaneously optimize pixel-level prediction accuracy and region-level segmentation consistency. The resulting loss function is defined as follows:

(29) $$L_{\mathrm{total}}=w_{b}\cdot L_{\mathrm{BCE}}+w_{d}\cdot L_{\mathrm{Dice}},$$

In the above formulation, $L_{\mathrm{total}}$ denotes the overall training loss, which is defined as a weighted combination of the Binary Cross-Entropy loss $L_{\mathrm{BCE}}$ and the Dice loss $L_{\mathrm{Dice}}$. The weighting coefficients $w_{b}$ and $w_{d}$ correspond to the contributions of the BCE loss and Dice loss, respectively, and are set to equal values in all experiments. The BCE loss is defined as:

(30) $$L_{\mathrm{BCE}}=-\frac{1}{N}\sum_{i=1}^{N}\left[y_{i}\log\left({\hat{y}}_{i}\right)+\left(1-y_{i}\right)\log\left(1-{\hat{y}}_{i}\right)\right],$$

Let *N* denote the total number of pixels in an input image. For the *i*-th pixel, $y_{i}$ represents the ground-truth binary label, where $y_{i}=1$ indicates a foreground pixel and $y_{i}=0$ indicates a background pixel. The predicted probability for the foreground class at the same pixel is denoted by ${\hat{y}}_{i}$, which is obtained from the network output after applying the sigmoid activation function. The Dice loss is computed as:

(31) $$L_{\mathrm{Dice}}=1-\frac{2\sum_{i}\left(y_{i}\cdot {\hat{y}}_{i}\right)+\epsilon }{\sum_{i}y_{i}+\sum_{i}{\hat{y}}_{i}+\epsilon },$$

In the Dice loss formulation, the summation is performed over all pixels in the image. The constant ϵ is introduced to ensure numerical stability and to prevent division by zero, and it is fixed to one throughout this work.

## 4. Experiments

### 4.1. Experimental Settings

#### 4.1.1. Datasets

We conduct extensive experiments on three widely used medical image segmentation benchmarks.

**ISIC2017** [38] is a large-scale dermoscopic image dataset released for skin lesion analysis. For the lesion segmentation task, it provides binary lesion masks that delineate lesion boundaries in dermoscopic images. The dataset contains 2000 training images, 150 validation images, and 600 test images.

**ISIC2018** [39] is another authoritative benchmark for dermoscopic lesion segmentation and classification. For the segmentation task, it consists of 2594 training images, 100 validation images, and 1000 test images. ISIC2018 offers higher image quality, more diverse lesion types, and more complex background variations, making it suitable for evaluating robustness and generalization under challenging clinical conditions.

**Kvasir-SEG** [40] is a colon polyp segmentation dataset collected using endoscopic devices, jointly released by Vestre Viken Hospital and Simula Research Laboratory. It includes 1000 images with pixel-level annotations, with image resolutions ranging from 332 × 487 to 1920 × 1072. The dataset presents substantial variations in brightness, contrast, texture, and polyp morphology, posing high demands on boundary perception and cross-domain generalization.

#### 4.1.2. Training Details

All competing methods are reproduced using their publicly available implementations and are retrained under the same data splits, input resolution, data augmentation, optimizer, learning-rate schedule, number of epochs, and evaluation code. The values reported in Table 1, Table 2 and Table 3 are obtained from these reproductions rather than copied directly from the original publications. Experiments are performed on a workstation equipped with an NVIDIA RTX 3080 GPU (16 GB VRAM) using the PyTorch 2.0 deep learning framework. The input images are uniformly resized to 256 × 256 pixels. Data augmentation techniques, including random flipping and random rotation, are employed to improve the model’s generalization ability. We adopt the AdamW optimizer with an initial learning rate of $1\times 10^{-3}$ and a weight decay of $1\times 10^{-2}$. To stabilize training, we employ the Cosine Annealing learning rate scheduler, which gradually reduces the learning rate to a minimum value $\eta_{\min}=1\times 10^{-5}$ with a cycle length $T_{\max}=50$. Training is conducted for 300 epochs with a batch size of 12. To evaluate robustness to random initialization and stochastic training variation, MGA-UNet is independently trained using three predefined random seeds, namely 42, 123, and 2026. The results of MGA-UNet in Table 1, Table 2 and Table 3 are reported as the mean ± sample standard deviation over these three runs. The competing methods are evaluated using seed 42 under the same data splits and experimental protocol.

#### 4.1.3. Evaluation Metrics

We evaluate binary segmentation using the macro-averaged foreground Intersection over Union (mIoU), Dice Similarity Coefficient (DSC), accuracy (AC), specificity (SP), and sensitivity (SE). Let TP, TN, FP, and FN denote the numbers of true-positive, true-negative, false-positive, and false-negative pixels, respectively. The metrics are defined as

(32) $$\mathrm{mIoU}=\frac{TP}{TP+FP+FN},\;\mathrm{DSC}=\frac{2TP}{2TP+FP+FN},$$

(33) $$\mathrm{AC}=\frac{TP+TN}{TP+TN+FP+FN},$$

(34) $$\mathrm{SP}=\frac{TN}{TN+FP},\;\mathrm{SE}=\frac{TP}{TP+FN}.$$

The network output is converted to a foreground probability map using the sigmoid function and binarized with a threshold of 0.5. Each metric is computed for each test image and then macro-averaged over the complete test set. A constant of $10^{-7}$ is added to each denominator in the implementation to avoid division by zero. If both the prediction and ground-truth mask are empty, the overlap score is defined as one.

### 4.2. Quantitative Results

To comprehensively evaluate the segmentation performance of the proposed MGA-UNet, we compare it with a diverse set of representative and recent methods on ISIC2018, ISIC2017, and Kvasir-SEG. The comparison covers CNN-based frameworks including U-Net and Residual U-Net, the attention-driven Att-UNet, Transformer-based models such as TransUNet and Swin-UNet, and recent Mamba-based approaches including VM-UNet, VM-UNetv2, and H-VMUNet. The quantitative results are summarized in Table 1, Table 2 and Table 3. For clarity, the best result for each metric is highlighted in red, while the second-best result is highlighted in blue.

**Figure 5:**
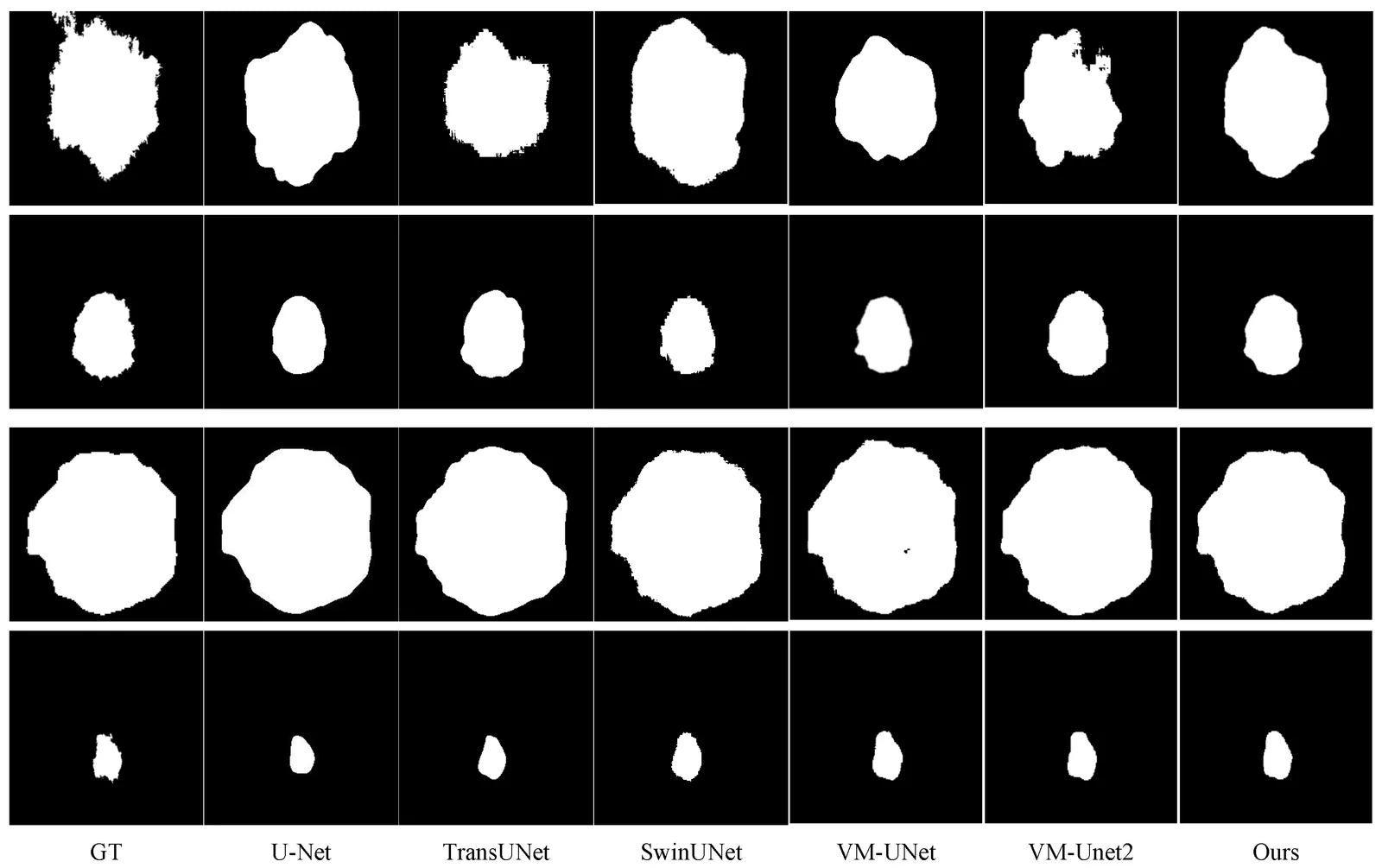
Qualitative comparisons of different methods on the ISIC2018 and ISIC2017 datasets. The segmentation results of different methods are presented for visual comparison. Best viewed with zoom-in.

On the ISIC2018 benchmark, MGA-UNet achieves competitive segmentation performance, with a mean mIoU of 86.79±0.07% and a mean DSC of 88.92±0.04%. Its mean DSC is numerically close to those of H-VMUNet (88.83%) and VM-UNetv2 (88.61%). MGA-UNet also records a mean accuracy of 95.13±0.08%, specificity of 97.12±0.15%, and sensitivity of 87.81±0.21%. Overall, the results demonstrate competitive lesion segmentation performance across the evaluated metrics (Figure 5).

The ISIC2017 dataset presents additional challenges due to a larger proportion of small lesions, low-contrast regions, and blurred boundaries. MGA-UNet achieves competitive segmentation performance, with a mean mIoU of 78.59±0.11% and a mean DSC of 88.01±0.07%. Its mean DSC is numerically close to those of H-VMUNet (87.81%) and VM-UNetv2 (87.67%). MGA-UNet also records mean accuracy, specificity, and sensitivity values of 96.06±0.03%, 97.94±0.06%, and 87.06±0.13%, respectively. These results indicate competitive lesion-region overlap and detection performance under structural ambiguity and background interference.

**Figure 6:**
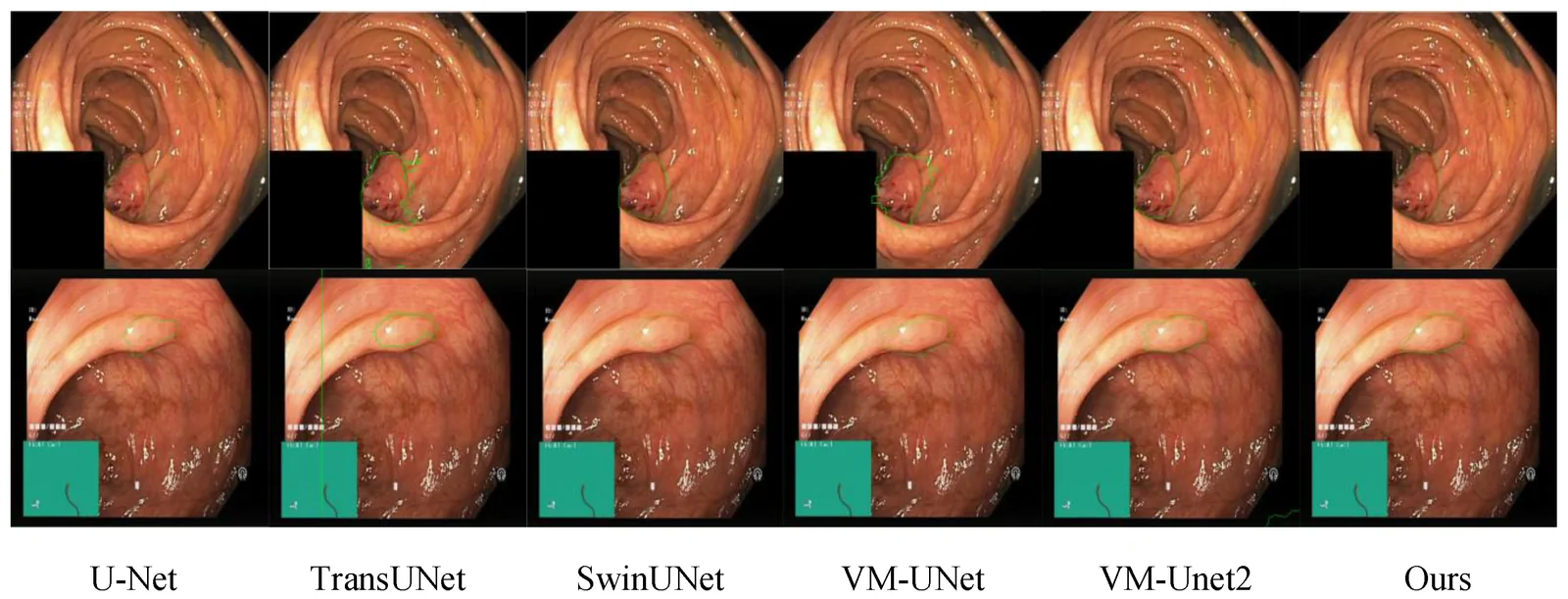
Qualitative comparisons of different methods on the Kvasir-SEG. For clearer visualization, the segmentation results of each method are highlighted in green on top of the original endoscopic images. Best viewed with zoom-in.

On the Kvasir-SEG dataset, which is characterized by substantial variations in illumination, color distribution, and polyp morphology, MGA-UNet achieves competitive segmentation performance, with mean mIoU, DSC, and sensitivity values of 75.84±0.08%, 85.91±0.04%, and 85.68±0.14%, respectively. Its mean DSC is numerically close to those of TransUNet (85.89%) and VM-UNetv2 (85.88%). MGA-UNet also records a mean accuracy of 95.78±0.09% and specificity of 97.42±0.12%. These results demonstrate competitive performance on Kvasir-SEG (Figure 6), while the small numerical differences between the strongest methods should be interpreted cautiously.

### 4.3. Model Complexity Analysis

To further assess model efficiency, we compare the number of parameters and FLOPs of different segmentation methods. As shown in Table 4, MGA-UNet contains 21.62 M parameters and requires 5.06 GFLOPs. Although its parameter count is not the lowest, its computational cost is substantially lower than that of most CNN- and Transformer-based methods and remains comparable to recent Mamba-based networks. These results demonstrate that MGA-UNet achieves a favorable balance between segmentation performance and computational efficiency.

### 4.4. Ablation Studies

To rigorously assess the contribution of each proposed component, we conducted a comprehensive set of ablation experiments on the ISIC2018, ISIC2017, and Kvasir-SEG datasets. Specifically, this subsection investigates the individual and combined effects of the three core modules, examines how different multi-scale depthwise convolution kernel configurations within GMAM influence segmentation performance, and analyzes the impact of various wavelet bases employed in WMB. Unless otherwise specified, the ablation experiments use random seed 42 and report single-run results; the multi-seed statistics in Table 1, Table 2 and Table 3 are used to evaluate the stability of the complete MGA-UNet model. For clarity, the best-performing results under each evaluation metric are highlighted in bold.

#### 4.4.1. Contribution of WMB, GMAM, and ASAM

We progressively removed the three key modules to evaluate their individual and combined effects. The results are reported in Table 5. As shown in Table 5, the baseline without any of the three modules yields the lowest performance, confirming the necessity of frequency-domain decomposition, multi-scale fusion, and top-*k* sparse global modeling. Introducing WMB improves performance owing to multi-frequency decomposition and selective modeling of high- and low-frequency features. Adding GMAM further enhances cross-scale semantic interaction and better fuses shallow spatial details with deep semantic information. It is observed that ASAM yields relatively modest performance gains on the ISIC2017 and ISIC2018 datasets. This behavior can be attributed to the fact that ASAM primarily operates on low-resolution bottleneck features and focuses on modeling long-range semantic dependencies rather than enhancing fine-grained boundary details. For skin lesion segmentation, where the lesion regions often exhibit relatively distinguishable color and texture patterns from the surrounding skin, segmentation performance is more strongly influenced by high-frequency detail modeling and effective multi-scale feature fusion, as provided by WMB and GMAM. In contrast, the Kvasir-SEG dataset presents more complex scenarios characterized by large variations in polyp size, shape, and global appearance, as well as cluttered background structures. In such cases, accurate segmentation relies more heavily on global contextual understanding and long-range semantic consistency. Consequently, ASAM demonstrates more pronounced improvements on Kvasir-SEG, highlighting its effectiveness in capturing global dependencies in structurally complex medical images. The best performance is achieved when all three modules are enabled, demonstrating their complementary and synergistic effects.

Beyond the individual improvements, it is noteworthy that the performance gains obtained by jointly enabling multiple modules are not strictly additive. For instance, combining WMB and GMAM leads to a larger improvement than applying either module alone, indicating a clear synergistic effect between frequency-domain decomposition and multi-scale feature aggregation. This suggests that frequency-aware representations extracted by WMB provide more informative inputs for cross-scale fusion in GMAM. Similarly, the combination of WMB and ASAM consistently outperforms single-module configurations, implying that frequency-enhanced features also facilitate more effective global semantic modeling in the bottleneck. When all three modules are activated, the network achieves the highest overall performance across datasets, demonstrating that local frequency modeling, multi-scale spatial fusion, and top-*k* sparse global attention play complementary roles rather than redundant ones.

#### 4.4.2. Impact of Multi-Scale Depthwise Convolution Kernels in GMAM

To investigate the multi-scale receptive field design in GMAM, various depthwise convolution kernel combinations were evaluated (Table 6).

Uniform kernel configurations provide limited receptive-field diversity and therefore cannot simultaneously emphasize fine boundary cues and broader contextual patterns. In contrast, the three parallel branches with 3×3, 5×5, and 7×7 depthwise kernels analyze the same aligned feature at complementary spatial scales. The 3×3 branch emphasizes local edges and textures, whereas the larger-kernel branches provide progressively broader contextual support. Aggregating these parallel responses before content-dependent gating yields the best segmentation performance, confirming the benefit of complementary multi-scale receptive fields rather than a sequential convolutional hierarchy.

#### 4.4.3. Influence of Wavelet Basis in WMB

Three common wavelet families were compared in WMB (Table 7). The Haar wavelet outperforms the others owing to its compact support and sharp edge response, making it particularly suitable for capturing high-frequency lesion details in medical images. Moreover, its simple and orthogonal basis facilitates stable feature decomposition and reconstruction, reducing sensitivity to dataset-specific texture variations. In contrast, more complex wavelet bases, such as Daubechies and Coiflet, may introduce additional smoothing or oscillatory artifacts, which can occasionally benefit specific datasets but tend to yield less consistent performance overall. These observations indicate that the Haar wavelet offers a favorable balance between representational capacity and robustness, thereby enhancing generalization across different medical image segmentation benchmarks.

Overall, the ablation studies confirm that each proposed component contributes to performance improvement from a distinct perspective. WMB enhances frequency-aware representations, GMAM strengthens multi-scale spatial interactions, and ASAM captures long-range semantic dependencies at the low-resolution bottleneck stage with controlled additional computational cost. When integrated together, these components form a unified framework that effectively addresses the intrinsic challenges of medical image segmentation, including scale variation, blurred boundaries, and complex global context.

#### 4.4.4. Effect of Operator Assignment in WMB

To further verify the rationale of the heterogeneous operator assignment in WMB (Table 8), we compare four combinations of SS2D and depthwise convolution for processing the low- and high-frequency components on the ISIC2018 dataset. Specifically, both frequency branches are processed using DWConv or SS2D, the proposed assignment is reversed, and the proposed configuration applies SS2D to the low-frequency branch and DWConv to the high-frequency branches. During this ablation study, all other network components, training settings, data splits, and wavelet configurations are kept unchanged to ensure a fair comparison.

## 5. Conclusions

MGA-UNet is a hybrid medical image segmentation framework that integrates frequency-domain modeling with state-space representations. The network combines wavelet-based frequency decomposition, gated multi-scale feature aggregation, and top-*k* sparse global context modeling to improve segmentation robustness under scale variation and boundary ambiguity. Experimental evaluations on public dermoscopic and endoscopic benchmarks demonstrate competitive segmentation performance across two sensor-captured medical imaging modalities. By jointly addressing acquisition-related appearance variations, ambiguous boundaries, and complex background interference, MGA-UNet provides an effective representation strategy for the computer-assisted analysis of images acquired by clinical imaging devices. Future work will extend the framework to volumetric medical images and further improve its computational efficiency. Potential directions include lightweight frequency representations, optimized state-space modules, and model compression techniques, such as knowledge distillation and structured pruning, to facilitate large-scale clinical deployment. Nevertheless, the current evaluation is limited to three public two-dimensional binary segmentation datasets and does not include cross-dataset transfer experiments or validation on an independent external clinical cohort. Therefore, the reported results should be interpreted as evidence of consistent performance across the evaluated benchmarks rather than proof of broad clinical or cross-domain generalization. Future work will evaluate MGA-UNet on additional imaging modalities, external clinical datasets, and cross-dataset settings.

## 6. Limitations

Despite its competitive performance, MGA-UNet has several limitations. First, the wavelet decomposition and reconstruction, SS2D operations, and top-k selection introduce additional implementation and hardware-dependent overhead compared with conventional convolutional architectures. Second, although the sensitivity analysis supports the default settings of ρ=0.5 and $k_{\min}=16$ , these fixed hyperparameters may not be optimal for all imaging modalities or feature resolutions. Third, the current evaluation is limited to three public two-dimensional binary segmentation datasets and does not include cross-dataset experiments or validation on an independent external clinical cohort. Therefore, further evaluation on additional imaging modalities, multiclass and volumetric segmentation tasks, and external clinical datasets is required.

## Figures and Tables

**Table 1:** Quantitative comparison on the ISIC2018 dataset (%). MGA-UNet is reported as mean ± sample standard deviation over three runs with random seeds 42, 123, and 2026.

Method	mIoU	DSC	AC	SP	SE
U-Net [3]	84.91	86.74	94.46	96.71	86.03
Att-UNet [16]	86.49	88.20	95.16	97.66	85.79
U-Net++ [4]	86.51	88.22	95.02	97.64	85.90
Residual U-Net [7]	85.09	86.89	94.68	96.88	86.59
TransUNet [34]	83.65	84.99	94.52	96.53	85.78
Swin-UNet [36]	84.84	86.57	94.53	97.42	83.71
VM-UNet [26]	85.35	88.52	94.47	96.86	85.15
VM-UNetv2 [41]	86.55	88.61	94.58	97.17	86.54
H-VMUNet [24]	86.47	88.83	94.55	96.30	86.09
MGA-UNet (Ours)	86.79±0.07	88.92±0.04	95.13±0.08	97.12±0.15	87.81±0.21

**Table 2:** Quantitative comparison on the ISIC2017 dataset (%). MGA-UNet is reported as mean ± sample standard deviation over three runs with random seeds 42, 123, and 2026.

Method	mIoU	DSC	AC	SP	SE
U-Net [3]	77.08	85.99	94.99	97.43	86.82
Att-UNet [16]	77.29	86.35	95.02	96.52	85.75
U-Net++ [4]	76.98	85.79	94.85	96.75	85.92
Residual U-Net [7]	77.48	86.52	95.84	97.05	86.54
TransUNet [34]	77.30	87.11	96.42	98.68	87.67
Swin-UNet [36]	77.22	86.78	96.02	97.67	85.78
VM-UNet [26]	76.97	86.98	95.72	97.78	85.48
VM-UNetv2 [41]	78.05	87.67	95.92	97.76	86.73
H-VMUNet [24]	77.86	87.81	94.35	97.04	86.51
MGA-UNet (Ours)	78.59±0.11	88.01±0.07	96.06±0.03	97.94±0.06	87.06±0.13

**Table 3:** Quantitative comparison on the Kvasir-SEG dataset (%). MGA-UNet is reported as mean ± sample standard deviation over three runs with random seeds 42, 123, and 2026.

Method	mIoU	DSC	AC	SP	SE
U-Net [3]	73.29	84.35	94.85	95.88	84.83
Att-UNet [16]	73.95	83.96	95.01	96.25	84.92
U-Net++ [4]	74.05	84.06	94.94	96.59	85.20
Residual U-Net [7]	73.89	83.65	93.68	96.47	84.87
TransUNet [34]	74.56	85.89	95.84	95.40	84.85
Swin-UNet [36]	75.29	85.66	94.81	96.93	83.73
VM-UNet [26]	75.01	84.93	94.85	96.25	84.98
VM-UNetv2 [41]	75.25	85.88	95.69	97.53	85.53
H-VMUNet [24]	75.36	85.41	94.52	97.24	85.61
MGA-UNet (Ours)	75.84±0.08	85.91±0.04	95.78±0.09	97.42±0.12	85.68±0.14

**Table 4:** Comparison of model parameters, computational complexity, and inference throughput. FPS is measured under the same hardware and inference settings with an input size of 256×256 and a batch size of 1.

Method	Parameters (M) ↓	GFLOPs ↓	FPS ↑
U-Net [3]	1.95	8.20	117.39
Att-UNet [16]	34.88	51.02	109.23
TransUNet [34]	105.28	80.68	71.59
Swin-UNet [36]	27.18	7.72	162.50
VM-UNet [26]	27.43	4.11	92.53
VM-UNetv2 [41]	17.91	4.40	98.71
**MGA-UNet (Ours)**	21.62	5.06	97.68

**Table 5:** Ablation study of the proposed modules on three datasets (DSC, %).

WMB	GMAM	ASAM	ISIC2018	ISIC2017	Kvasir-SEG
×	×	×	88.52	86.98	84.93
✓	×	×	88.64	87.06	85.34
×	✓	×	88.56	87.20	85.08
×	×	✓	88.65	86.99	85.16
✓	✓	×	88.90	87.80	85.81
×	✓	✓	88.69	87.61	85.76
✓	×	✓	88.78	87.86	85.54
✓	✓	✓	**88.95**	**87.97**	**85.96**

**Table 6:** Ablation on depthwise convolution kernel sizes in GMAM (DSC on ISIC2018, %).

Branch 1	Branch 2	Branch 3	DSC
DWConv 3 × 3	DWConv 3 × 3	DWConv 3 × 3	88.57
DWConv 5 × 5	DWConv 5 × 5	DWConv 5 × 5	88.63
DWConv 7 × 7	DWConv 7 × 7	DWConv 7 × 7	88.58
DWConv 3 × 3	DWConv 5 × 5	DWConv 5 × 5	88.79
DWConv 3 × 3	DWConv 5 × 5	DWConv 7 × 7	**88.95**

**Table 7:** Performance of different wavelet bases in WMB (DSC on datasets, %).

Wavelet Family	ISIC2018	ISIC2017	Kvasir-SEG
Haar	**88.95**	**87.97**	**85.96**
Daubechies	88.72	87.83	85.75
Coiflet	88.75	87.69	85.01

**Table 8:** Ablation study of different operator assignments in WMB on ISIC2018.

Low-Frequency Branch	High-Frequency Branch	DSC (%) ↑	GFLOPs ↓
DWConv	DWConv	85.42	9.05
SS2D	SS2D	86.21	4.24
DWConv	SS2D	86.24	7.24
SS2D	DWConv	**88.95**	**5.06**

## Data Availability

The datasets analyzed in this study are publicly available. The ISIC 2017 and ISIC 2018 datasets can be obtained from the official International Skin Imaging Collaboration archive, and the Kvasir-SEG dataset can be obtained from its official public repository.
